# MRI reporter genes in the era of gene transfer

**DOI:** 10.1002/ctm2.1135

**Published:** 2022-12-05

**Authors:** Dan Cohen, Hyla Allouche‐Arnon, Amnon Bar‐Shir

**Affiliations:** ^1^ Department of Nuclear Medicine Tel‐Aviv Sourasky Medical Center Tel Aviv Israel; ^2^ Department of Molecular Chemistry and Materials Science Weizmann Institute of Science Rehovot Israel

## 1 ARE WE LIVING IN THE ERA OF GENE TRANSFER?

The development of genetically engineered therapeutic cells and the evolution of the field of gene transfer seem to attract more and more attention as advanced approach with which to overcome the challenges that are inaccessible to conventional therapies.^1^ One specific example is the introduction of chimeric antigen receptor T (CAR‐T) cells into clinical practice that has revolutionized the field of cancer immunotherapy. These therapeutic cells, genetically engineered to recognize a specific antigen expressed on target cells, have produced robust responses in patients with B‐cell malignancies,^2^ and efforts are being made to translate this success to solid tumours as well.^3^ However, serious toxicities, such as the immune effector cell‐associated neurotoxicity syndrome, have been associated with this therapy, with, as yet, not fully understood mechanisms. As the field of gene transfer evolves, improved in vivo gene tracking techniques are necessary to monitor and assess the efficacy and safety of these novel interventions. Using imaging modalities routinely employed in clinical practice to this end constitutes a challenging opportunity. If developed properly and implemented successfully, such 3D imaging techniques to spatially locate the expression of a gene reporter can revamp cells and gene therapy protocols with a vision to enable real‐time assessment of therapy progression or failure. With the recent implementation of a billion mRNA vaccine doses for coronavirus disease (COVID‐19), with no or negligible harmful effects,^4^ the taboo of introducing transgenes in humans and the accompanying psychological barriers have been lifted. While this success has accelerated the development of mRNA vaccines against tumour‐associated or tumour‐specific antigens,^5^ it has also led to the understanding that the use of genetic engineering strategies in clinical setups could change the way we will fight incurable diseases and monitor the efficacy of advanced therapeutics.

## 2 FROM CLINICAL MOLECULAR IMAGING TO ADVANCED IMAGING OF GENE EXPRESSION

### 2.1 Clinical molecular imaging

In contrast to conventional morphologic‐anatomic imaging techniques, *molecular imaging* techniques aim to spatially locate injected, imageable molecules. In positron emission tomography (PET) imaging, the most common molecular imaging technique practiced in clinical oncology, the 3D image acquired represents the whole‐body distribution of an administrated molecule‐of‐interest labelled with a positron‐emitting isotope. This way, for example, when 2‐deoxyglucose labelled with ^18^F isotope (i.e., FDG) is intravenously injected, PET detects a radioactive signal in foci characterized by glucose hypermetabolism (where the injected FDG is ‘trapped’).^6^

### 2.2 Imaging (reporter) gene expression

For almost three decades, scientists have also been exploring clinical imaging modalities for the non‐invasive mapping of gene expression, using pairs of *reporter genes* and *reporter probes*.^7^, ^8^ *Reporter genes*, extensively used in basic research practices, are genes that are inserted into cells‐of‐interest. *Reporter probes* are imageable molecules, which undergo enzymatic conversion and, in some cases, also cellular ‘trapping’ upon the expression of their paired *reporter genes*. Implementing *reporter genes* to visualize therapeutic cells following their administration to patients, using a clinically used imaging technology, would change the way we assess the efficacy and toxicity of cell‐based therapies with capabilities to spatially map the viability, distribution, localization and even quantities of the administrated cells.

## 3 REPORTER GENES IN CLINICAL SETUPS: THE HSV1‐*tk* CASE

The herpes simplex virus type‐1 thymidine kinase (HSV1‐*tk*) gene represents one extensively studied reporter gene.^9^ This gene encodes for the expression of the HSV1‐TK enzyme, which phosphorylates both natural and synthetic deoxyribonucleosides (dNs). Synthetic radio‐labelled dNs, detectable by PET, can function as *reporter probe*s if they undergo cellular ‘trapping’ upon phosphorylation by HSV1‐TK. After injecting such a *reporter probe*, the expression of the HSV1‐*tk* reporter gene can be monitored. Indeed, in 2009, an ^18^F‐labelled dN (^18^F‐FHBG: 9‐[4‐[^18^F]fluoro‐3‐(hydroxymethyl)butyl]guanine) was used as a reporter probe for PET imaging of a human patient treated with therapeutic cells genetically engineered to target glioblastoma cells and express the HSV1‐*tk* reporter gene.^10^ In this case, the injected *reporter probe* successfully detected two brain foci where the infused cells had localized, validating that corresponding morphologic imaging findings represented *viable* therapeutic cells homing to *active* malignant sites.

## 4 THE HSV1‐*tk* AS AN MRI REPORTER GENE: WE NEED AN MRI‐DETECTABLE PROBE

While PET imaging benefits from very high sensitivity, it does not provide anatomical information, and spatial localization of the therapeutic cells expressing the *reporter gene* requires hybrid systems, such as PET‐CT (computed tomography) or PET‐MRI (magnetic resonance imaging). Moreover, PET‐detectable probes, which emit radioactivity that decays over time, pose challenges in terms of safety as well as accessibility, handling and production. Realizing these drawbacks, we have demonstrated the ability to map the expression of the HSV1‐*tk* reporter gene with an MRI‐detectable *reporter probe*.^11^ To this end, a synthetic dN, the thymidine‐analogue, 5‐methyl‐dihhydrothymidine (5‐MDHT),^12^ was found to be a specific substrate for the HSV1‐TK enzyme, thus accumulating, upon its phosphorylation, in cells engineered to express HSV1‐*tk*. Moreover, 5‐MDHT was rationalized to be an ideal MRI *reporter probe* that is detected, without the need for labelling, by a technique known as *chemical exchange saturation transfer* (CEST).^13^ This makes 5‐MDHT an ideal reporter probe for imaging HSV1‐*tk* reporter gene expression with CEST‐MRI.

## 5 APPLYING CEST‐MRI TO MAP REPORTER PROBES: WE CAN DO IT WITH COLOURS!

In basic MRI techniques, radiofrequency waves known to excite tissue water protons are applied at their specific resonance frequency (*ω*). Then, as the excited water protons realign with the constant external magnetic field, a current is generated in a receiver antenna, and recording its per‐voxel intensity constitutes the MRI contrast. In the CEST‐MRI^13^, ^14^ technique, a ‘tagging’ radiofrequency wave is added to a conventional MRI acquisition protocol to magnetically label the exchangeable protons of a CEST *reporter probe*. This proton pool, with a resonance frequency Δ*ω*, different than that of water protons (*ω*), spontaneously transfers its ‘tagged’ magnetization to the surrounding water through a dynamic proton exchange phenomenon. Measuring the change in water magnetization after this ‘tag’ transfer (i.e., the CEST signal) enables the 3D localization of the *reporter probe* (i.e., 5‐MDHT) that ‘reports’ on the localization of the *reporter gene* (i.e., HSV1‐*tk*). Importantly, CEST agents require no paramagnetic labels (in contrast to traditional gadolinium‐labelled MRI agents), thus opening the opportunity to use synthetic and bioorganic molecules as *reporter probes*. Capitalizing on this feature, we have demonstrated that another synthetic dN, pyrrolo‐deoxycytidine (pdC), which generates strong CEST contrast as well, can be used as a *reporter probe* to image the expression of the *reporter gene* encoding to the enzyme *Drosophila melanogaster* deoxyribonucleoside kinase (*Dm*‐dNK).^15^

**FIGURE 1:**
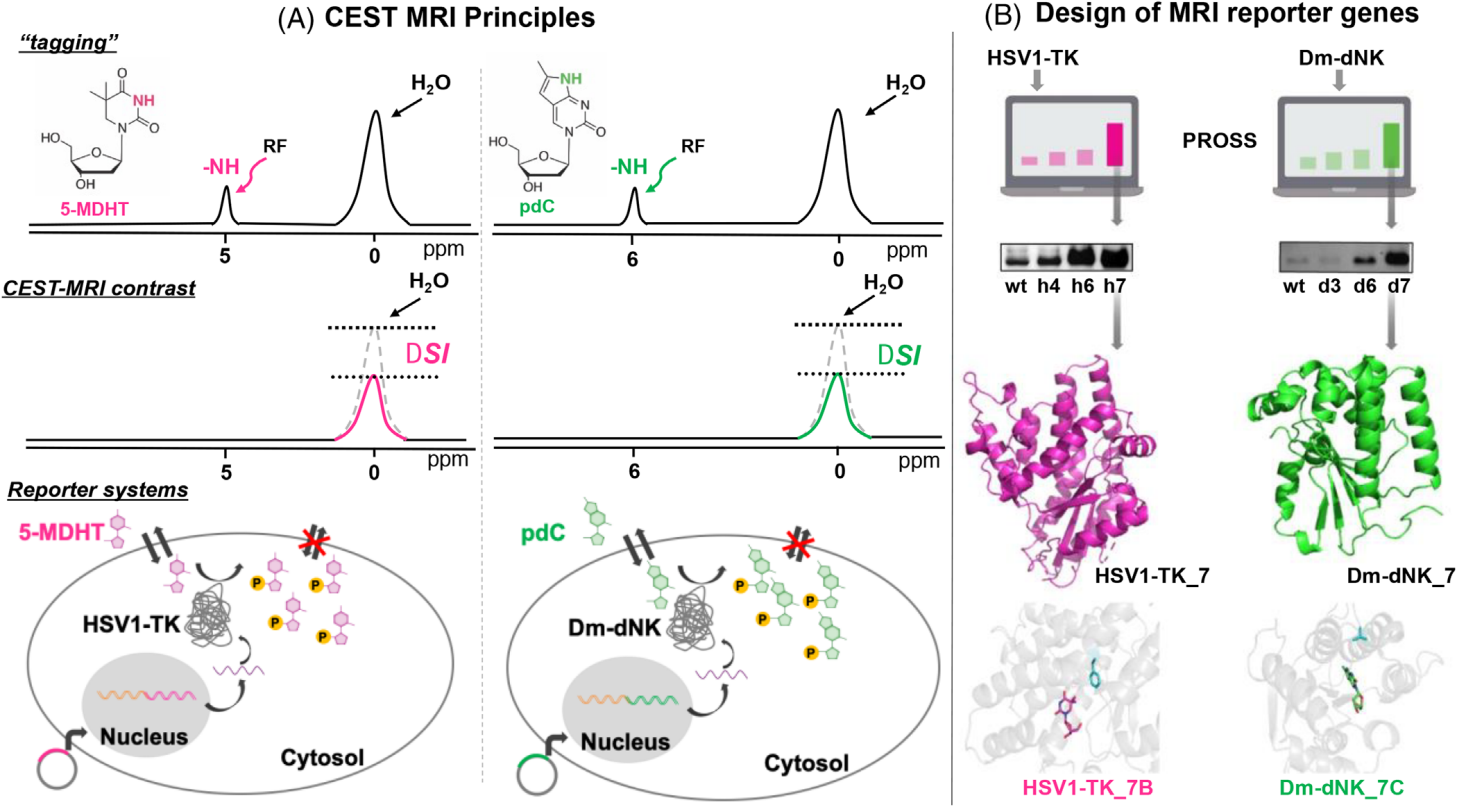
(A) The principle of chemical exchange saturation transfer (CEST) magnetic resonance imaging (MRI) and the mode of action of the *reporter probe*/*reporter gene* system. Applying selective ‘tagging’ pulses at the frequency of the ‐NH exchangeable protons of 5‐MDHT (5 ppm, left) or pdC (6 ppm, right) results in a reduction of the surrounding water signal, ΔSI. This change in the MRI signal (i.e., contrast) can be assigned with magenta and green colours for CEST generated by 5‐MDHT at 5 ppm and pdC at 6 ppm, respectively. A schematic illustration of the *reporter gene*/*reporter probe* systems used is shown in which 5‐MDHT accumulates in HSV1‐TK‐expressing cells and pdC accumulates in Dm‐dNK‐expressing cells upon their phosphorylation. (B) The design of highly active and orthogonal HSV1‐*tk* and *Dm‐dNK* reporter genes. Following automated PROSS design of both enzymes, the Western blot analysis of the output enzymes shows higher expression levels compared to their wild‐type analogue. The ultimate PROSS‐designs, HSV1TK_7 (20 surface mutations) and Dm‐dNK_7C (15 surface mutations), were further mutated at their active site to improve their activity and orthogonality to obtain HSV1‐TK_7B (specifically binds and converts 5‐MDHT) and Dm‐dNK_7C (specifically binds and converts pdC).

As the CEST‐MRI contrast is ‘turned on’ on demand, only upon the application of a frequency‐specific, probe‐selective ‘tagging’, multiple CEST *reporter probes* reactive to different radiofrequency pulses (Δω_1_, Δω_2_, Δω_3_, …) can be administered simultaneously and then mapped and presented in a pseudo‐colour manner. For example, the two CEST‐reactive *reporter probes*, 5‐MDHT and pdC, whose ‐NH exchangeable protons have different Δ*ω* of Δ*ω*_1_ = 5 ppm and Δ*ω*_2_ = 6 ppm, respectively, could be used to generate artificial colours in CEST‐MRI maps (Figure 1A). That is, one colour is assigned to the CEST signal generated by 5‐MDHT (Δ*ω*_1_ = 5 ppm, magenta) and another colour is assigned to the CEST signal generated by pdC (Δ*ω*_2_ = 6 ppm, green), with the potential to spatially localize the expression of two reporter genes, HSV1‐*tk* and *Dm*‐dNK, respectively.

## 6 OPTIMIZING THE EXPRESSION OF ENGINEERED REPORTER GENES

Generally speaking, engineered *reporter genes* exhibit low levels of heterologous expression, limiting the sensitivity for *reporter probe* detection upon imaging, particularly (but not only) with MRI. To overcome this, we have applied a fully automated computation approach named PROSS,^16^ a structure‐ and evolution‐based protein‐design method, to improve the performance of MRI *reporter genes*. PROSS is very attractive for the development of clinically relevant *reporter genes*, because this approach requires the experimental examination of only a few (3–5) proposed mutants, thus overcoming the lack of high‐throughput screening capabilities of PET‐ and MRI‐based platforms. For example, a PROSS‐designed HSV1‐TK (namely, HSV1‐TK_7) exhibiting 20 mutations on its surface, showed a more than six‐fold increase in expression levels compared to the wild‐type HSV1‐TK (Figure 1B, top). As no mutation is introduced by PROSS into the active site, the obtained HSV1‐TK_7 variant can be readily used in PET imaging (with ^18^F‐FHBG, for example). Importantly, PROSS resulted in an improved cellular tolerability of HSV1‐TK_7 (and Dm‐dNK_7) with enhanced accumulation of the reporter probes 5‐MDHT (and pdC). Further, site‐directed mutagenesis was applied to obtain the optimized HSV1‐TK_7B and Dm‐dNK_7C as ultimately orthogonal *reporter genes* for the 5‐MDHT and pdC *reporter probes*, respectively (Figure 1B, bottom).

## 7 IN VIVO DUAL‐COLOUR MRI MAPPING OF EXPRESSED GENETICALLY ENGINEERED TRANSGENES

**FIGURE 2:**
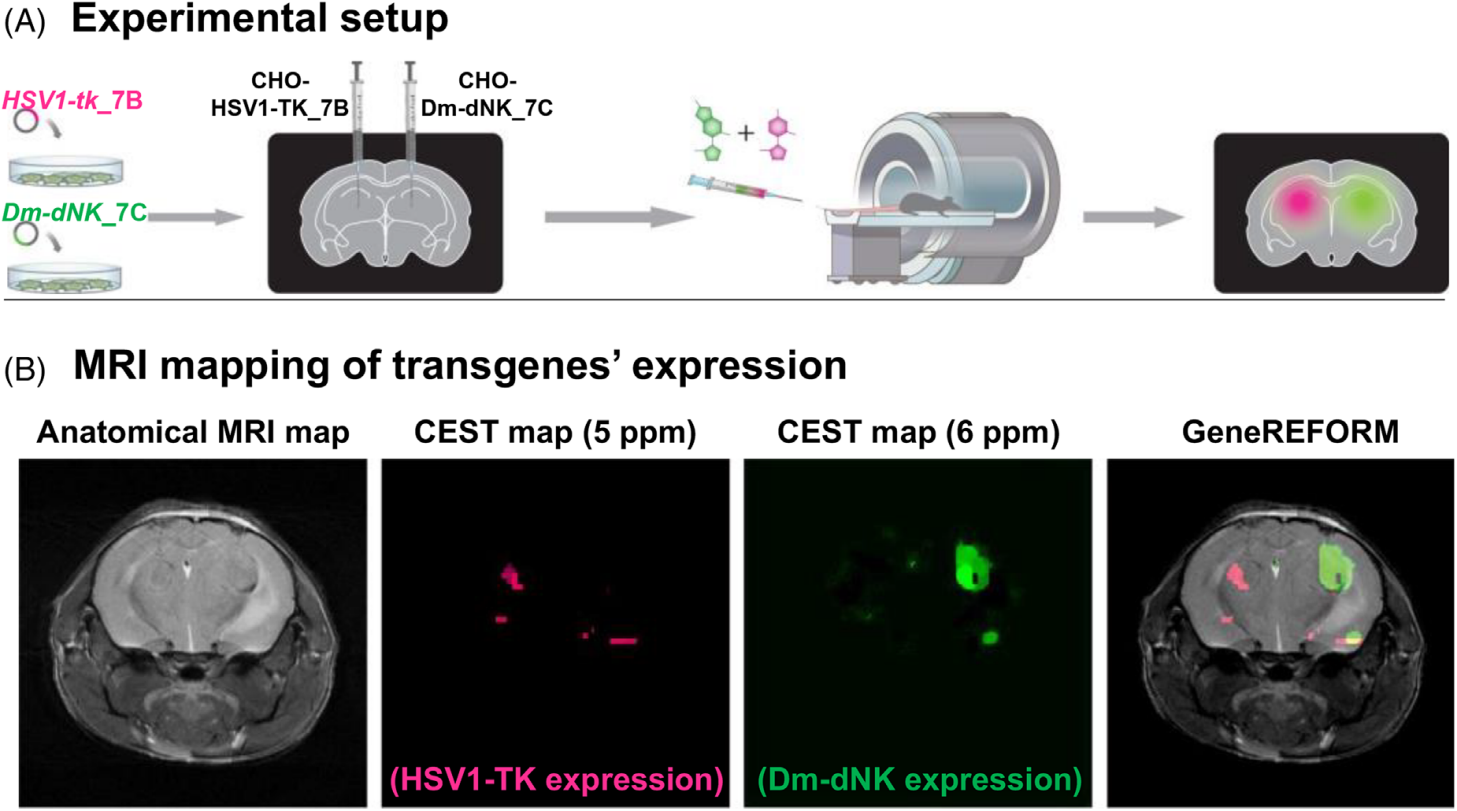
In vivo magnetic resonance imaging (MRI) of GeneREFORM. (A) Schematic presentation of the experimental setup. CHO cells were genetically engineered to express either HSV1‐TK_7B or Dm‐dNK_7C followed by intracranial injection into two hemispheres of the examined mice. After the development of CHO^HSV1‐TK_7B^ and CHO^Dm‐dNK_7C^ tumours in the brain, a mixture of 5‐MDHT and pdC is intravenously injected and chemical exchange saturation transfer (CEST) data are acquired to differentiate between the two types of genetically engineered tumours. (B) An in vivo MRI of a representative examined mouse. From left‐to‐right: An anatomical view of the mouse brain, CEST‐MRI at 5 ppm (representing 5‐MDHT accumulation in HSV1‐TK_7B‐expressing cells), CEST‐MRI at 6 ppm (representing pdC accumulation in Dm‐dNK_7C‐expressing cells) and the pseudo‐coloured CEST map of the implanted tumours overlaid on the corresponding anatomical image.

Having established this dual‐colour, MRI‐detectable reporter system, which we named GeneREFORM (genetically engineered reporters for multicolour MRI),^17^ we decided to examine the ability to map the two 5‐MDHT/HSV1‐TK_7B and pdC/Dm‐dNK_7C *reporter probe*/*reporter gene* pairs in the same subject. HSV1‐TK_7B and Dm‐dNK_7C were expressed in tumour cells that were transplanted into the brains of examined mice. Developing genetically engineered tumours in two hemispheres of the mice brains, the studied subjects were intravenously administered with a mixture of 5‐MDHT and pdC *reporter probes* (Figure 2A, top). CEST‐MRI enabled mapping of the transgenes’ expression and the spatial localization of the two types of tumour cells in a pseudo‐multicolour fashion, overlaid on a conventional anatomical MRI map (Figure 2B). Of note is that on PET imaging, in comparison, with simultaneous injection of two different molecules labelled with positron‐emitting‐isotopes (either the same labelling isotope or another), a focus of signal detection will indicate the accumulation of one of the molecules or both, but differentiation between the sources is impossible.

## 8 CONCLUSION

With data accumulated for decades to support the strengths provided by the ever‐evolving field of preclinical and clinical molecular imaging, the era of gene transfer opens the opportunity to concentrate efforts on imaging of gene expression. The ability to map genetically engineered transgenes with clinically translatable setups, such as MRI, would enable clinicians to monitor and study the efficacy and toxicity of cell and gene therapy. Not only that, it may also provide direct data on cellular viability and quantity, potentially minimizing the need for the indirect data that are currently utilized in the clinic. The multidisciplinary scientific progress made in the fields of *reporter genes* and MRI‐detectable *reporter probes*,^17^, ^18^, ^19^ as well as in cutting‐edge MRI techniques, could together pave the way to ultimately provide a clinically applicable diagnostic MRI framework with which to image *reporter genes*. Given the already known strengths provided by MRI in clinical anatomic and functional imaging, MRI *reporter genes* add cellular and molecular imaging capabilities to this modality. The demonstration of MRI *reporter gene*s in other clinically relevant setups, such as oncolytic viral therapy,^20^ and the fact that multiple *reporter gene*s can be now simultaneously mapped with MRI and presented in a multicolour fashion,^17^ should make MRI a single imaging modality that could stand at the forefront of the gene transfer revolution.

## CONFLICT OF INTEREST

The authors declare that there is no conflict of interest.
